# Chains Stiffness Effect on the Vertical Segregation of Mixed Polymer Brushes in Selective Solvent

**DOI:** 10.3390/polym15030644

**Published:** 2023-01-27

**Authors:** Ivan V. Lukiev, Yana A. Mogelnitskaya, Ivan V. Mikhailov, Anatoly A. Darinskii

**Affiliations:** 1 Center for Chemical Engineering, ITMO University, 197101 St. Petersburg, Russia; 2 Institute of Macromolecular Compounds, Russian Academy of Sciences, 199004 St. Petersburg, Russia

**Keywords:** mixed polymer brushes, height switch, phase segregation, selective solvent

## Abstract

The microstructure of the binary polymer brushes in the selective solvent was studied using the numerical lattice self-consisting field approach. The case was considered when the selectivity to the solvent (the Flory–Huggins parameter χ) was varied only for one type of chains (responsive chains) while the others (non-responsive chains) remained hydrophilic (χ = 0). In such a brush, with an increase in the hydrophobicity of the responsive chains, a transition occurs between two two-layer microstructures. In the initial state the ends of the longer responsive chains are located near the external surface of the brush and those of non-responsive chains are inside the brush. When the hydrophobicity of the responsive chains becomes high enough then the reversed two-layer microstructure is formed, when the ends of non-responsive chains are located near the brush surface and the responsive chains collapse on the brush bottom. In contrast to previous works, the stiffness parameter (Kuhn segment length *p*) for one or for both types of chains was varied and its effect on the mechanism and characteristics of the transition was studied. If the stiffness of only responsive chains increases, then the transition occurs with the formation of an intermediate three-layer microstructure, where a layer of responsive chains is located between layers formed by non-responsive ones. If both types of chains have the same *p*, then the transition occurs gradually without the formation of an intermediate three-layer microstructure. For both cases, the effect of *p* on the critical value of $\chi^{*}$, corresponding to the transition point and on the steepness of the transition was investigated.

## 1. Introduction

The design of advanced materials that can adapt their properties to the environment changes (so-called smart materials) is one of the key issues of modern nanotechnology. Among such materials, an important place is occupied by smart surface coatings. Polymer brushes are considered to be promising systems for the creation of such coatings. Being able to improve product performance and reduce maintenance expenses [1], smart polymer coatings (SPC) can be exploited in a variety of industrial sectors, providing a wide range of applications such as switch sensors [2,3,4], antifouling surfaces [5,6,7,8], lubrication [9], targeting drug delivery [10,11,12,13,14,15], and chromatographic protein separations [16,17,18].

The external trigger that controls the SPC response can be either temperature [19], changes in electric [20] and magnetic fields [21], ionic strength [22], exposure to light or radiation [23,24], pH shift [25], or the addition of antigen [26] or enzyme [27]. Thereby, SPC are able not only to drastically alter their physical properties, but also to perform as a susceptible sensor of environmental changes.

A common approach to the implementation of SPC materials is the creation of binary brushes from chemically distinct macromolecules. The macromolecules may have different affinity to each other and/or to solvent molecules. This difference usually leads to the microphase segregation inside the brush. By the change of the affinity (for example, due to the replacement of the solvent by another or by temperature change), polymer chains can switch their positions. This molecular switch is capable of undergoing a prompt conformational transition, which is similar to a first-order phase transition [28,29,30].

Correspondingly, the morphology of the brush changes depends on its composition, chemical structure of chains forming the brush, grafting density, etc. Extreme cases of segregation include lateral and vertical ones. If the brush components are highly incompatible, lateral segregation arises. Alternatively, if the compatibility between the brush components is high enough, vertical segregation occurs [31,32,33,34,35]. The greatest interest for smart coating creation is mixed brushes with vertical microsegregation, which implies that macromolecules of one type are located inside the brush near the grafting surface, and that macromolecules of another type form the periphery of the brush. The brush morphology can change due to environmental changes: internal chains appear on the surface, and external chains go inside. This behavior can radically change the chemical composition of the surface, and as a result, alter the properties of nanocoating. The choice of components for SPC based on binary brushes is determined both by the purposes of their use and their response to changes in external conditions. In particular, macromolecules that can be utilized for biological and medical purposes are of big interest as their qualities play vital roles in cell adhesion, growth, differentiation, morphogenesis, and proliferation [5,36]. The ability to perform an environment-depending switching is determined by the physical and chemical properties of the polymer chains in the brushes. One of the most widely used polymers that meet these conditions is Poly(N-isopropylacrylamide) (PNIPAAm). This temperature-sensitive polymer was used for the responsive brushes construction while combined with other macromolecules such as polystyrene (PS) [37], poly(acrylic acid) (PAA) [38,39], poly(ethylene glycol) (PEG) [40,41], or antimicrobial peptides [42]. PNIPAAm exhibits a lower critical solution temperature (LCST) close to the physiological temperature of humans (∼31 °C) [43], which makes its use in biomedical coatings promising.

There is a large number of theoretical and experimental works devoted to the study of binary brushes [31,32,39,40,43,44,45,46,47,48,49,50,51,52,53]. Depending on parameters such as the grafting density, solvent selectivity, length ratio, brush composition, and polymer incompatibility, a binary brush can form different phases such as a disordered phase, in which both polymer species are mixed, or ripple and dimple phases, in which the polymers exhibit a lateral and/or vertical segregation. In this work we will consider brushes with vertical segregation. Among the works devoted to the theory and computer modeling of such brushes, Refs. [39,40,47,51,54] should be noted. In these works, segregation occurred when the brush was placed in a selective solvent and the transition between different morphologies arose due to the solvent change. By using coarse-grained models, different characteristics were calculated, such as the transition point, transition width, and transition barrier. It should be noted that in all these works it was assumed that both components of the brush consist of chains with the same flexibility. At the same time, the actual values of the Kuhn lengths for chains in real binary brushes can differ by five or more times (for instance, 0.68 nm for PEG and 4 nm for PNIPAAm).

The purpose of this work was to fill this gap. We also focused on the binary brushes in the selective solvent but considered the case when brush components had different flexibility. Our main goal was to estimate how this difference affected the segregation and the conformation transition of the chains in the brush. As the calculation method we used the numerical lattice variant of the self-consistent field method developed by Scheutjens and Fleer [55]. This method was applied successfully for many polymer systems and, in particular, for the polymer brushes [56,57,58,59,60].

The outline of the paper is as follows. The model and the method are described in the Section 2. The Section 3 contains the results of the calculation and their discussion. The conclusions on the results are presented in the Section 4.

## 2. Model and Method

### 2.1. Model

**Figure 1:**
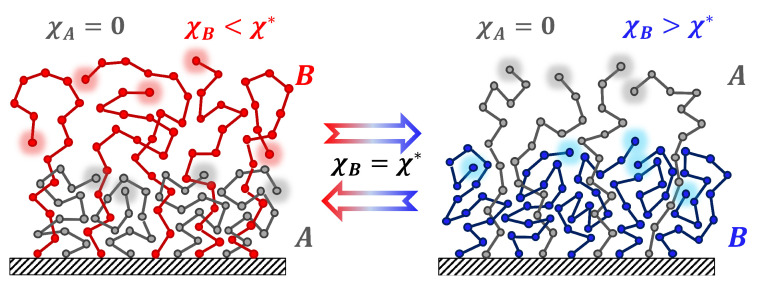
Schematic representation of vertical phase segregation in a binary polymer brush consisting of two components, *A* and *B* ($\sigma_{A}=\sigma_{B}$, $N_{A}<N_{B}$).

A coarse-grained model was used to study vertical phase segregation in mixed polymer brushes (Figure 1). Each brush was constructed of two types of polymer chains with a different solvent selectivity being densely grafted onto an impenetrable flat surface. Different types of grafted chains have different behavior depending on the solvent molecules. One type of chains (denoted as type *A*) is solvaphilic and independent from the strength of solvent selectivity. The second type of chains (denoted as type *B*) is sensible to the solvent selectivity.

According to the Flory theory, free energy of interactions $F_{int}$ between different components can be described in each point of the system as the product of their volume fractions φ by the corresponding Flory–Huggins parameter χ:

(1) $$F_{int}=\chi_{AB}\varphi_{A}\varphi_{B}+\chi_{A}\varphi_{A}\varphi_{s}+\chi_{B}\varphi_{B}\varphi_{s},$$

where $\varphi_{s}$ is the volume fraction of solvent molecules, $\varphi_{A}$ and $\varphi_{B}$ are the volume fractions of monomer units of grafted chains. In our model, the solvent is athermal to type *A* chains ($\chi_{A}=0$). There is no additional interaction between grafted chains of types *A* and *B* ($\chi_{AB}=0$). The solvent quality in relation to type *B* chains was varied. Thus, the strength of the solvent selectivity is characterized by using a single parameter $\chi_{B}$, which is hereafter denoted as χ for simplicity. The free energy of the Flory–Huggins interactions is

(2) $$F_{int}=\chi \varphi_{B}\varphi_{s}$$

It is assumed that all monomer units in the chain are identical and have the same linear size *a*. The value of *a* is used as the unit length. The grafting density of the chains is characterized by the dimensionless ratio $\sigma =a^{2}/s$, where *s* is an average surface area per one grafted macromolecule. It is further assumed that the grafting density of the different types of chains is the same: $\sigma_{A}=\sigma_{B}=\sigma$.

The polymer chain of each type *A* and *B* contains $N_{A}$ and $N_{B}$ monomer units. The stiffness of the chains is determined by the length of the Kuhn segment $p_{A}$ and $p_{B}$.

The segregation transition point is taken to be the value of the solvent selectivity strength at which the first moments of the free ends distribution of both chains become equal.

### 2.2. Self-Consistent Field Method

To investigate the properties of the binary brushes, we use the self-consistent field approach. All possible interactions between the components of a molecular system within the framework of this method are taken into account by means of the averaged chemical potential *u*. Such an approximation simplifies the simulation of a set of polymer chains to the simulation of single chains in the field of an average chemical potential. This technique saves significant computing resources.

In the present work we use the lattice numerical variant of SCF method developed by Scheutjens and Fleer (SF-SCF method) [55,61]. The simulation is carried out on a discrete cubic lattice consisting of cells with an edge length equal to *a*. It is assumed that the grafted chains overlap quite strongly and form a relatively uniform brush. So the density distribution of the monomer units in the planes parallel to the grafting surface is considered to be uniform, therefore all characteristics of a flat polymer brush, such as the polymer volume fraction φ and the effective chemical potential *u*, have a gradient only along the *z* axis oriented perpendicular to this surface. All parallel layers, starting from the grafting surface, are numbered from z=0 to $z_{max}$. The grafting surface has the number z=0, all grafted chain ends are fixed in the layer z=1.

When polymer chains walk on the lattice, it is assumed that some monomer units may overlap, i.e., occupy the same lattice node. However, this artifact is suppressed by the high chemical potential that arises in this case and prevent effectively through the incompressibility condition, which is to be fulfilled in each layer:

(3) $$\sum_{X}\varphi_{X}\left(z\right)=1,\;\;\;X=A,B,s$$

Hereinafter, the subscripts indicate that the parameter belongs to the polymer chain of the type *A*, type *B*, or to the solvent (*s*) molecule, respectively.

The chain stiffness is characterized by the Kuhn segment length *p*, which obeys the equation [62]:

(4) $$\frac{p}{a}=\frac{1+\langle \cos\gamma \rangle }{1-\langle \cos\gamma \rangle },$$

where γ=π−θ is the supplementary angle to the valence angle θ.

In this model, the correlation between two consecutive chain links is given by the potential:

(5) $$\frac{U^{ang}\left(\gamma \right)}{k_{B}T}=K\left(1-\cos\gamma \right),$$

where *K* is a coefficient of the bonds correlation.

On the cubic lattice, two consecutive bonds can have three relative orientations: (1) straight conformation, where a bond makes an angle θ=π with the preceding one (that corresponds to γ=0 and $U_{\left(s\right)}^{ang}=0$); (2) perpendicular kink (γ=π/2 and $U_{\left(p\right)}^{ang}=K$)); (3) backfold “fracture” (γ=π and $U_{\left(b\right)}^{ang}=2K$). The weighting factor $\lambda_{s}$ of the straight conformation and the weighting factor $\lambda_{b}$ of the backfold conformation are related to the weighting factor $\lambda_{p}$ of the perpendicular kink as

(6) $$\lambda_{s}=\lambda_{p}\exp\left(-\frac{U_{\left(s\right)}^{ang}}{k_{B}T}\right)/\exp\left(-\frac{U_{\left(p\right)}^{ang}}{k_{B}T}\right)=\lambda_{p}\exp\left(K\right),$$

(7) $$\lambda_{b}=\lambda_{p}\exp\left(-\frac{U_{\left(b\right)}^{ang}}{k_{B}T}\right)/\exp\left(-\frac{U_{\left(p\right)}^{ang}}{k_{B}T}\right)=\lambda_{p}\exp\left(-K\right)$$

The sum of the weighting factors in the case of cubic lattice obeys the normalization condition:

(8) $$\lambda_{s}+4\lambda_{p}+\lambda_{b}=1$$

The average cosine of the angle γ can be calculated by the equation:

(9) $$\langle \cos\gamma \rangle =\lambda_{s}\cos0+4\lambda_{p}\cos\frac{\pi }{2}+\lambda_{b}\cos\pi =\lambda_{s}-\lambda_{b}$$

According to the above Equations (4) and (6)–(9), weight coefficient $\lambda_{p}$ can be found by the formulae:

(10) $$\lambda_{p}={\left(\exp K+\frac{1}{\exp K}+4\right)}^{-1},$$

where

(11) $$\exp K=\sqrt{{\left(1-\frac{p}{a}\right)}^{2}+\frac{p}{a}}+\frac{p}{a}-1$$

Weight coefficients ($\lambda_{b}$,$\lambda_{p}$, $\lambda_{s}$) for different orientations of neighboring segments along the chain can be expressed through the length of the Kuhn segment *p* using the Formulas (6), (7), (10) and (11).

The grafted chains are immersed in solvent. The free energy per unit area of the grafting surface is calculated as the energy of Flory–Huggins interactions $F_{int}$ and the sum of the negative logarithms of the partition functions $Q_{X}$ of all components (X=i,s) in a constant effective external fields $u_{X}\left(z\right)$ minus the work of these fields $\sum_{z}u_{X}\left(z\right)\varphi_{X}\left(z\right)$ (according to the Legendre transform):

(12) $$F\left\{u,\varphi \right\}=F_{int}\left\{\varphi_{X}\left(z\right)\right\}-k_{B}T\sum_{X,X\neq s}\sigma_{X}\ln Q_{X}\left\{u_{X}\left(z\right)\right\}-\sum_{z}\sum_{X}u_{X}\left(z\right)\varphi_{X}\left(z\right),$$

where $\sigma_{X}$ is the *X*-chain grafting density.

The Flory–Huggins energy, which characterizes the repulsion between monomer units and solvent molecules, is described by the equation:

(13) $$\frac{F_{int}}{k_{B}T}=\chi \sum_{z}\varphi_{B}\left(z\right)\left(\langle \varphi_{s}\left(z\right)\rangle -\varphi_{s}^{\infty }\right)+\varphi_{s}\left(z\right)\left(\langle \varphi_{B}\left(z\right)\rangle -\varphi_{B}^{\infty }\right),$$

where χ is the Flory–Huggins parameter, $\varphi_{B}\left(z\right)$ and $\varphi_{s}\left(z\right)$ are the volume fractions of the thermosensitive polymer and solvent, respectively, $\varphi_{B}^{\infty }=0$ and $\varphi_{s}^{\infty }=1$ are the bulk volume fractions at an infinite distance from the grafting surface. The angle brackets denote the averaging of the volume fraction in the layer *z* over neighboring layers.

The main target of the SCF modeling is to find the polymers volume fraction distributions corresponding to the minimum of free energy under the condition of incompressibility. To perform this, the following functional is minimized:

(14) $$F=F\left\{u,\varphi \right\}+\sum_{z}\alpha \left(z\right)\left[\sum_{X}\varphi_{X}\left(z\right)-1\right],$$

where α(z) is the Lagrange field (set of Lagrange multipliers). The Lagrange field α(z) corresponding to the minimum of functional F Equation (14) is calculated during the iterative procedure of the gradient descent:

(15) $$\alpha \left(z\right)\leftarrow \alpha \left(z\right)+\eta \frac{\delta F}{\delta \alpha (z)}=\alpha \left(z\right)+\eta \left[\sum_{X}\varphi_{X}\left(z\right)-1\right],$$

where η<1/2 is the convergence step size, which corresponds to the highest convergence rate.

Minimization of F with respect to the volume fractions $\varphi_{X}\left(z\right)$ allows us to calculate the potential fields $u_{X}\left(z\right)$:

(16) $$\frac{\delta F}{\delta \varphi_{X}\left(z\right)}=0\;\;\;\Rightarrow \;\;\;u_{X}\left(z\right)\leftarrow \alpha \left(z\right)+\frac{\delta F_{int}\left\{\varphi_{X}\left(z\right)\right\}}{\delta \varphi_{X}\left(z\right)}$$

Minimization of F with respect to the potential fields $u_{X}\left(z\right)$ provides a way to calculate the volume fraction distributions $\varphi_{X}\left(z\right)$:

(17) $$\frac{\delta F}{\delta u_{X}\left(z\right)}=0\;\;\;\Rightarrow \;\;\;\varphi_{X}\left(z\right)\leftarrow \sigma_{X}\frac{\delta (-\ln Q_{X})}{\delta u_{X}\left(z\right)}$$

The numerical solution of the Equations (16) and (17) is carried out using a special iterative procedure that uses special matrices-propagators to calculate the partition functions of polymer chains. The conformations of macromolecules are considered as Markov chains of the second order. A similar implementation of the Markov formalism for simulation of the semi-rigid polymer chains was proposed in the fundamental works of Refs. [56,57,58,59]. The algorithm of the procedure used in this work is described in details in our previous work [60]. Special software [63] was developed to release the above simulation.

## 3. Results and Discussion

### 3.1. Profiles of the Polymer Volume Fractions and Free Ends Distributions

**Figure 2:**
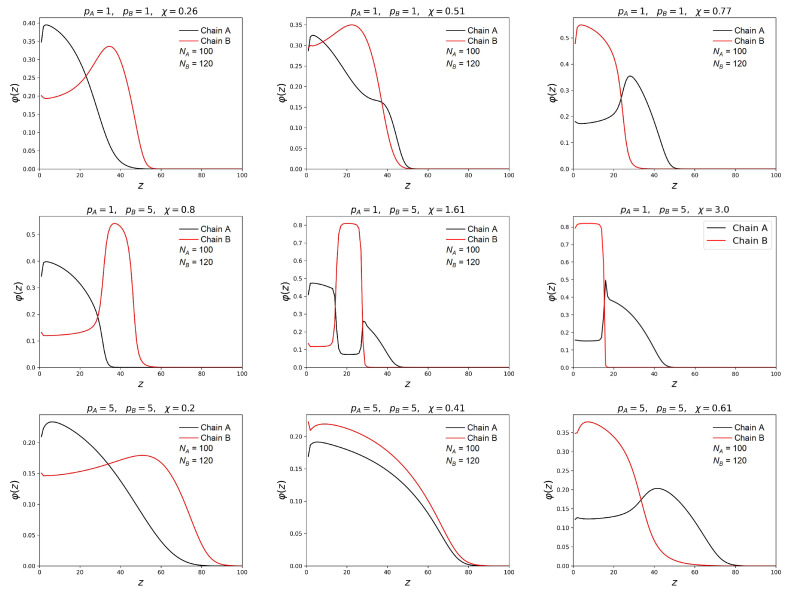
Volume fraction profiles of the monomer units of chains in the brush at the grafting density σ=0.1. Profiles for non-responsible chains are denoted in black, and responsible ones are denoted in red. The distributions in the middle column correspond to the segregation transition point $\chi =\chi^{*}$. In neighboring columns, the solvent selectivity differ towards the smaller and the higher values, respectively.

**Figure 3:**
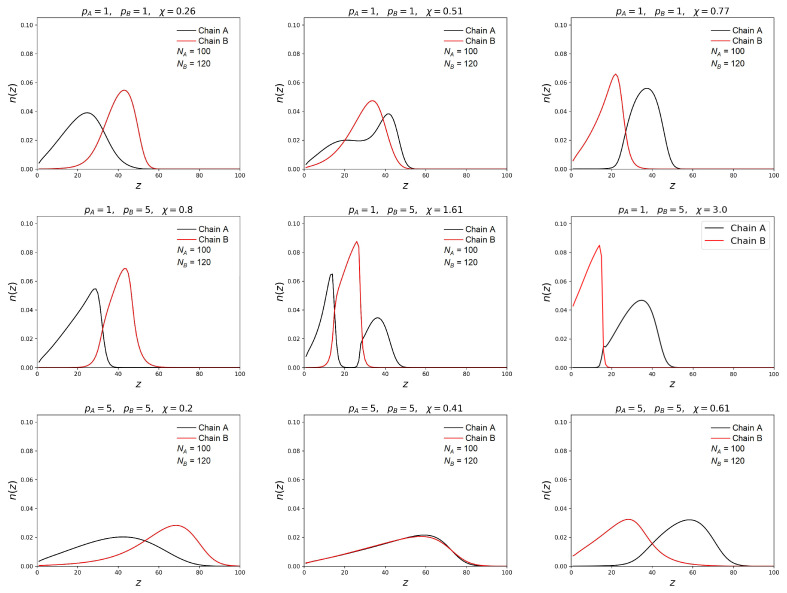
Distribution profiles of the chain free ends in the binary brushes. The notations are the same as in the previous figure.

Figure 2 and Figure 3 show how the change of the solvent selectivity affects the segregation in the mixed brush.

The profiles of volume fractions φ(z) and free ends distribution n(z) for both types of chains are presented for three different brush compositions: (1) both chains are flexible $p_{A}=p_{B}=1$; (2) the responsible chain is semi-rigid and the non-responsible one is flexible $p_{B}>p_{A}=1$; (3) both chains are semi-rigid $p_{A}=p_{B}>1$. In all cases chain A was shorter than chain B. For all brushes at small values of the parameter χ, the two layer structure with a different concentration of chains is observed. The layer located near the grafting surface is enriched in short chains, and the outer layer is enriched in much longer chains. When both chains have the same flexibility by an increase of the parameter χ, it leads to a smooth change of the volume density distributions positions. The terminal monomers also change their location: the ends of shorter chains come to the surface, and the ends of the longer responsive chains go inside the brush. These results are in qualitative agreement with the results of SCF and MD simulation obtained by other authors for mixed brushes consisting of flexible chains [40,51].

A different behavior is observed when the responsive chains are semi-rigid, and the shorter chains are flexible. In this case, the transition from initial two-layer structure to the reversed final two-layer structure goes through the formation of the intermediate three-layer structure, when the layer enriched with the A-monomer units lies between the two B-monomer-rich layers. Correspondingly, the responsive B-chains are divided into two populations. Part of them is located in the lower layers, and another part is in the upper layers. With the increase of χ, the intermediate B-rich layer is shifted as a whole to the grafting surface wherein the non-responsive flexible chains are “squeezed out” from the bottom layer into the outer layer. This results in the formation of the three-layer structure of the A-B-A type, where the middle layer is represented by terminal units of longer chains, and both the lower and upper layers are occupied by the ends of short chains.

When chain A is flexible and chain B is semi-rigid, similar changes in the profiles are observed, as in the first case, even in a more pronounced form. It should be noted that the three-layer structure remains in a wider range of χ values close to the transition point. With a considerable solubility decrease of longer chains they collapse strongly, forming a dense layer near the grafting surface. When this layer is compressed, short chains “leave” it. In this case, shorter chains are more strongly stretched and lose their conformational entropy, and longer chains minimize the free energy of polymer-solvent interaction by reducing the contact of monomer units with solvent molecules. In a denser near-wall layer, there is a competition for space between shorter and longer chains. As the selectivity of the solvent increases, short chains “leave” the near-wall layer, giving way to longer chains.

### 3.2. Dependence of the Transition Point on the Brush Characteristics

For binary brushes consisting of longer responsive chains and shorter non-responsive ones, as we have shown above, the increase of the parameter χ for responsive circuits leads to the transition between two types of two-layer structures. In the initial structure, the lower layer is enriched with non-responsive chains, and the upper one with responsive chains. In the final structure, the layers are arranged in reverse order. This transition occurs in some range of χ which depends on the brush characteristics: chain lengths $N_{A}$ and $N_{B}$, grafting density σ and Kuhn segment lengths $p_{A}$ and $p_{B}$. For the quantitative description of this transition we introduce the transition point, i.e., the value of $\chi^{*}$, when the average heights of both types of chains become equal to each other: $H_{A}=H_{B}=H^{*}$. The average height is defined as the first moment of the distribution of free ends for a given type of chain:

(18) $$H_{X}=\frac{\sum_{z}n\left(z\right)z}{\sum_{z}n\left(z\right)},\;\;\;X=A,B$$

**Figure 4:**
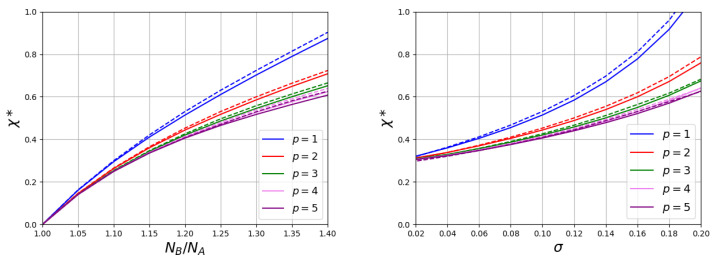
The values of transition points $\chi^{*}$. (**The left picture**) shows the impact of the ratio of polymerization degrees of responsive and non-responsive chains at σ=0.1. (**The right picture**) shows the impact of grafting density σ at $N_{B}/N_{A}=1.2$. The polymerization degree of non-responsive polymer is varied: $N_{A}$ = 100 (solid lines) and $N_{A}$ = 200 (dashed lines). Kuhn lengths of both chains change simultaneously: $p=p_{A}=p_{B}$.

**Figure 5:**
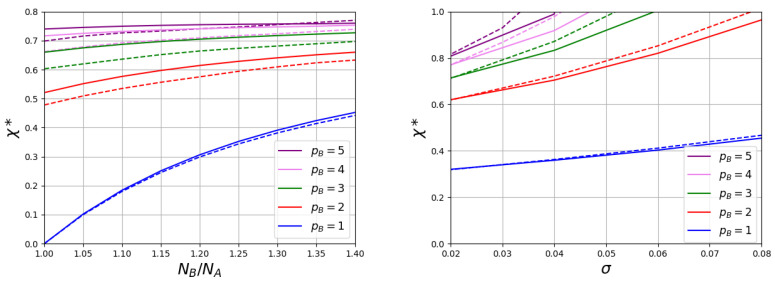
The values of transition points $\chi^{*}$. (**The left picture**) shows the impact of the ratio of polymerization degrees of responsive and non-responsive chains at σ=0.01. (**The right picture**) shows the impact of grafting density σ at $N_{B}/N_{A}=1.2$. The polymerization degree of non-responsive polymer is varied: $N_{A}$ = 100 (solid lines) and $N_{A}$ = 200 (dashed lines). Kuhn lengths of responsive polymer is varied $p_{B}$ and constant for non-responsive polymer $p_{A}=1$.

Figure 4 and Figure 5 demonstrate the dependencies of the transition point $\chi^{*}$ on the ratio $N_{B}/N_{A}$ and on the grafting density σ for brushes with different stiffness *p* of grafted macromolecules.

**Figure 6:**
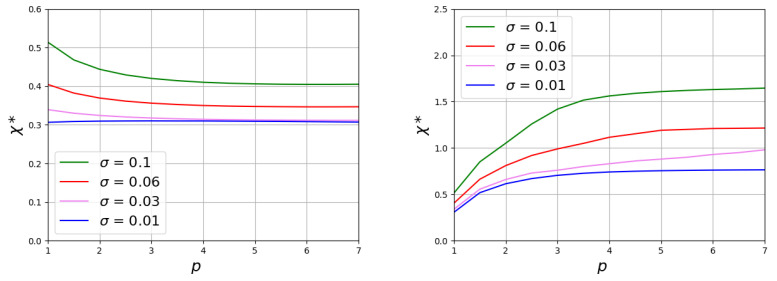
Solvent selectivity strength values as a function of Kuhn segment length of chains. In the (**left figure**), the stiffness of both chains changes simultaneously $p=p_{A}=p_{B}$. In the (**right figure**), only the stiffness of the long responsible chains $p=p_{B}$ increases, while chains A remain flexible $p_{A}=1$. The given data corresponds to the value of ratio $N_{B}/N_{A}=1.2$.

At a fixed chain grafting density, the value of the transition point $\chi^{*}$ increases with an increase in the ratio $N_{B}/N_{A}$. An increase of σ also leads to growth of $\chi^{*}$. When the values of Kuhn segment are the same for both chains $p_{A}=p_{B}=p$, these functions remain universal in a wide range of the polymerization degrees of the non-responsive chains (at $N_{A}=100$ and $N_{A}=200$ the curves practically coincide). For the same values of $N_{B}/N_{A}$ and σ, the simultaneous increase in the Kuhn segment length for both types of chains significantly reduces the value of the transition point. The type of function $\chi^{*}\left(p\right)$ depends on the grafting density (Figure 6 the left graph). At a large σ, the curve shows an initial region of decline and at a large *p* it reaches a constant value. The region of the initial decay decreases with the decrease of σ and almost disappears at low grafting density.

Let us now consider the case when non-responsive chains remain flexible $p_{A}=1$, but with the Kuhn length $p_{B}$ for responsive chains changes. An example of the structure changing of such a brush when changing χ is shown in Figure 2 and Figure 3. In Figure 5, the dependencies of the values of the transition points $\chi^{*}$ on the ratio of the polymerization degree of short and long chains and the grafting density are shown for brushes consisting of flexible chains A ($p_{A}=1$) and long chains B with various stiffness. As in previous case, the value of the transition point increases both with $N_{B}/N_{A}$ and with the grafting density σ. However, the effect of an increase of *p* occurs to be opposite to that in the first case. At the same values of $N_{B}/N_{A}$ and σ, the transition point grows with $p_{B}$. It should also be noted that in this case the obtained curves are not invariant with respect to ratio $N_{B}/N_{A}$. These dependencies cease to be invariant to the ratio $N_{B}/N_{A}$. At the same value of this ratio an increase of the A-chain length leads to a decrease in the value of $\chi^{*}$.

Both for brushes consisting of chains with the same stiffness and for brushes consisting of flexible and semi-rigid chains, the effect of the stiffness on $\chi^{*}$ becomes weaker with growth of *p* (Figure 6).

### 3.3. Steepness of the Transition

**Figure 7:**
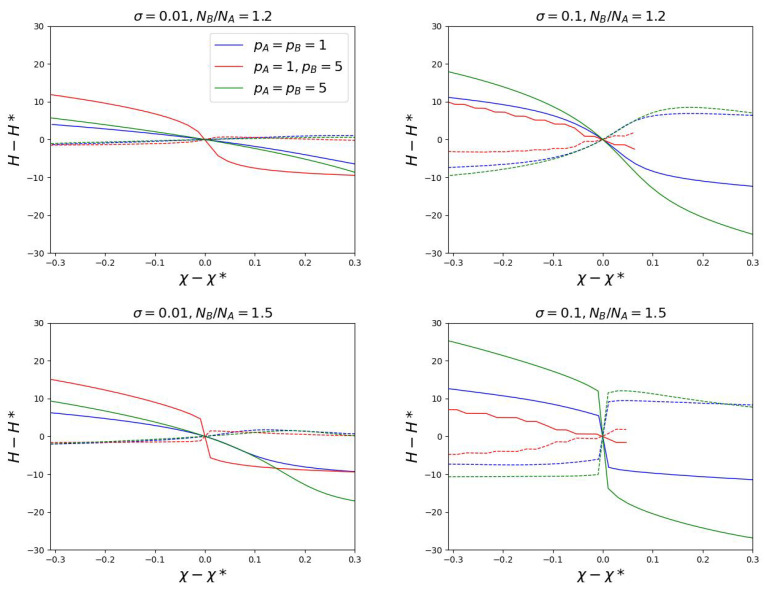
The first moments of the free ends distributions for chains A and B as a function of the solvent selectivity. Dashed lines indicate non-responsible chain A, solid lines indicate responsible chain B.

From a practical point of view, it is important to predict not only the value of $\chi^{*}$ in the transition point, but also the steepness of the transition. Greater steepness means greater sensitivity of the brush to changes in external conditions (for example, temperature or solvent changes). Figure 7 shows how the deviations $\left(H_{A}-H^{*}\right)$ and $\left(H_{B}-H^{*}\right)$ of the heights of each component from the value of $H^{*}$ in the transition point depend on the deviation of χ from the transition value $\chi^{*}$. Results for the brushes consisting of chains with equal $p_{A}=p_{B}$ and with different $p_{A}=1,\;p_{B}>1$ Kuhn segments are shown. It is seen that the transition sharpness depends differently on the brush parameters at low and large grafting density. At low σ the maximum steepness is observed for chains with different stiffness. For chains with the same *p*, the transition is continuous without a jump. For a large σ, the opposite behavior is observed. The transition is continuous in the brushes where the semi-rigid responsive chains are mixed with flexible non-responsive ones. When $p_{A}=p_{B}=p$ some steepness appears, which increases with the growth of *p*. In both cases the steepness is larger for brushes with a larger ratio $N_{B}/N_{A}$. Thus, two strategies can be proposed for preparing mixed brushes that are sensitive to small changes in external conditions. If the polymer components are characterized by close rigidity, it is necessary to prepare brushes with a high grafting density. If the brush consists of flexible and semi-rigid chains the best is a brush with a low grafting density.

## 4. Conclusions

By using the numerical lattice self-consisting field method, the structure of the mixed brush consisting of two types of chain was considered. The chains composing the brush had different affinity for the solvent, which was characterized by the value of the Flory–Huggins parameter χ. The case was studied when the value of this parameter was varied for one type of chains (responsive chains), and was remained unchanged for another (non-responsive chains). It was also assumed that chains of different types remain compatible with each other for all χ. In contrast to previous works, the stiffness parameter (Kuhn segment length) for responsive or for both chains was varied. It was shown that by an increase of χ there was a transition from the initial two-layer structure, where the non-responsive chains were on the bottom and responsive ones on the top of the brush, to the reversed two-layer one, where responsive chains were on the brush bottom. The value of the Flory–Huggins parameter $\chi^{*}$ (transition point), when the ends of both types of chains were at the same height relative to the grafting surface, was calculated. The dependencies of $\chi^{*}$ on the different brush parameters were obtained. The effect of the variation of the stiffness parameter on the value of $\chi^{*}$ was studied for two cases: (1) the brushes consist of chains with the same stiffness and (2) the stiffness parameter is varied only for the responsive chains and does not change for the non-responsive component. In the first case the transition point $\chi^{*}$ decreases as the stiffness of both chains grows. In the second case an increase of *p* for the responsive chains leads to an increase of the transition parameter $\chi^{*}$. An analysis of the dependence of the transition steepness on the system parameters showed that an increase in the chain stiffness of one or both components of the brush can lead to an increase in its sensitivity to small changes in external conditions. In the first case, this effect was manifested in brushes with a low grafting density. For mixed brushes of the second type, it was optimal to use brushes with a high grafting density.

In this paper, we have limited ourselves to considering binary brushes containing the same number of chains of both types $\sigma_{A}=\sigma_{B}$, because, as a rule, the grafting densities of chains in real binary brushes are really close in magnitude. Our other significant model assumption was that the non-responsive solvatophilic chains did not change its solvent affinity, when the solvent selectivity to the responsive chains changed. Real polymers have their own individual dependencies of solubility on temperature and have different sizes of the monomer units. In our modeling, we do not pretend to directly compare the obtained data with real polymer systems, however, we have shown that chain stiffness can play a key role in the parameterization of coarse-grained models. In this work, the dynamics of the segregation transition and the phenomenon of mixed and lateral segregation for chains with poor compatibility were also not considered, because such calculations can be performed only using other dynamic simulation 3D-methods.

The issues raised form the prospect of continuing this work and can be a direction for future investigations.

## Data Availability

Not applicable.

## Abbreviations

The following abbreviations are used in this manuscript:

LCST	The lower critical solution temperature
MD	Molecular dynamics
PAA	Poly(acrylic acid)
PEG	Poly(ethylene glycol)
PNIPAAm	Poly(N-isopropylacrylamide)
PS	Polystyrene
SCF (method)	The self-consistent field (method)
SF-SCF (method)	The Scheutjens–Fleer self-consistent field (method)
SPC	Smart polymer coatings
